# Activation of mammalian target of rapamycin (mTOR) in triple negative feline mammary carcinomas

**DOI:** 10.1186/1746-6148-9-80

**Published:** 2013-04-15

**Authors:** Lorella Maniscalco, Yolanda Millán, Selina Iussich, Mauro Denina, Raquel Sánchez-Céspedes, Francesca Gattino, Bartolomeo Biolatti, Nobuo Sasaki, Takayuki Nakagawa, Maria Flavia Di Renzo, Juana Martín de las Mulas, Raffaella De Maria

**Affiliations:** 1 University of Turin, Department of Veterinary Sciences, via L. da Vinci 44, 10095 Grugliasco, Italy; 2 University of Córdoba, Department of Comparative Pathology, Faculty of Veterinary Medicine, Carretera Madrid-Cádiz km 369, 14014 Córdoba, Spain; 3 Veterinary Surgery, Graduate School of Agricultural and Life Sciences, the University of Tokyo,1-1-1 Yayoi, Bunkyo, Tokyo 113-8657, Japan; 4 University of Torino Medical School, Department of Oncological Sciences at the Institute for Cancer Research and Treatment SP 142, Km. 3.95, 10060 Candiolo, Turin, Italy

## Abstract

**Background:**

Triple negative breast cancer (TNBC) in humans is defined by the absence of oestrogen receptor (ER), progesterone receptor (PR) and HER2 overexpression. Mammalian target of rapamycin (mTOR) is overexpressed in TNBC and it represents a potential target for the treatment of this aggressive tumour. Feline mammary carcinoma (FMC) is considered to be a model for hormone-independent human breast cancer. This study investigated mTOR and p-mTOR expression in FMC in relation to triple negative (TN) phenotype.

**Results:**

The expression of mTOR, p-mTOR, ERα, PR and HER2 was evaluated in 58 FMCs by immunohistochemistry and in six FMC cell lines by Western blot analysis. 53.5% of FMC analyzed were ER, PR, HER2 negative (TN-FMC) while 56.9% and 55.2% of cases expressed mTOR and p-mTOR respectively. In this study we found that m-TOR and p-mTOR were more frequently detected in TN-FMC and in HER2 negative samples.

**Conclusions:**

In this study, we demonstrate that there is also a FMC subset defined as TN FMC, which is characterised by a statistically significant association with m-TOR and p-mTOR expression as demonstrated in human breast cancer.

## Background

Triple negative breast cancer (TNBC) in humans is a distinct subset of breast cancer that is defined by the lack of immunohistochemical (IHC) expression of the oestrogen receptor (ER) and progesterone receptor (PR) and a lack of human epidermal growth factor receptor 2 (HER2) overexpression [1]. This subtype of cancer comprises 15-20% of patients with breast cancer for which targeted therapy is currently unavailable [2,3]. To develop new targeted therapies for this subset of tumours, researchers focused their attention on the different intracellular cell signaling pathways responsible for tumour growth, invasion and metastasis in TNBC [4]. One of the most pathways studied is the PI3K/AKT/mTOR pathway, which can be activated by different membrane tyrosine kinase receptors, including the epidermal growth factor (EGFR) family of growth receptors, insulin-like growth factor receptor (IGF-R) and ERα [5].

A key downstream component of the PI3K pathway is the mammalian target of rapamycin (mTOR), a serine/threonine kinase involved in tumour formation and progression [3]. mTOR is activated by phosphorylation at Ser2448 via the PI3K kinase/AKT signalling pathway and is autophosphorylated at Ser2481 [6]. mTOR has two main downstream messengers, the ribosomal p70 S6 kinase (S6K1) and the eukaryotic translation initiation factor 4E-binding protein (4E-BP1) [7]. The activation of S6K1 and 4E-BP1 by mTOR induces mRNA translation and a subsequent increase in protein synthesis that is essential for cell growth and proliferation [8,9].

Based on its role in tumour formation and progression, targeted therapy against mTOR has been shown to decrease tumour growth in model systems [10,11] and several mTOR inhibitors, such as everolimus, deforolimus and temsirolimus, have been used in clinical trials for the treatment of multiple cancer types including breast cancer [12].

Recently, it has been reported that the activated form of mTOR, phospho-mTOR (p-mTOR), detected at nuclear level, was expressed more frequently in triple negative (TN) human breast cancers compared with non-TN cancers [3], suggesting that mTOR may play a more important role in the progression of TNBC and could be considered a new target for the treatment of this tumour sub-type [9,13-15].

Feline mammary carcinoma (FMC) shares many biological and molecular similarities with human breast cancer [16] and is considered an excellent model for aggressive, hormone-independent human breast cancers overexpressing HER2 [17-19]. The percentage of FMCs that are negative for ER and PR range from 37% [20] to 54.2% [21]. Regarding HER2 expression in feline mammary carcinomas scientific data are controversial. Some authors [19,22,23] showed that HER2 is expressed from 39% to 56.3% which is similar to human breast cancer (20-30%) [24] while Rasotto and colleagues [25] showed HER2 expression in only the 5% of analyzed cases. We have recently found that AKT is expressed in FMCs, and its expression correlated with poor prognosis [22], suggesting a role for the PI3K/AKT/mTOR pathway in FMC pathogenesis.

The aim of this study was to investigate the role of mTOR and p-mTOR in feline mammary tumours and cell lines with regard to the TN FMC status and clinical outcome to understand the role of mTOR in feline mammary tumour progression and to evaluate whether the feline model may be considered the first animal model for the TNBC.

## Methods

### Tissue samples and histology

Tissue samples from 58 cases of FMC, 18 benign lesions and 8 normal mammary tissues were collected from the 2000–2008 archives of the Diagnostic Laboratories of the Department of Veterinary Sciences of the University of Turin, Italy, and the Department of Animal Pathology, University of Córdoba, Spain. Details of age, breed, ovariectomy status and tumour size data were retrieved from the hospital database. Postoperative clinical, radiological and echographic examinations at 6, 12, 18 and 24 months after surgery were performed by veterinarians to detect the presence of distant organ metastases or the local recurrence of the primary tumour. Animals that died due to mammary carcinoma were submitted for post-mortem examination to confirm the pathological diagnosis; subjects that died of non-tumour-related causes (confirmed by necroscopy) during the follow-up period were excluded from the study. The disease-free interval (DFI) was considered to be the number of days between surgery and tumour recurrence and/or evidence of metastatic disease while the overall survival (OS) was considered the period between surgery and animal death. Normal mammary tissue used as control were collected from necroscopy (after owners’ consensus) of female cats that were neither spayed nor treated with hormonal therapy during their life and died spontaneously at the Veterinary Hospital of the University of Turin.

The samples were fixed in 4% neutral buffered formalin, paraffin embedded, sectioned at 4 μm and stained with haematoxylin and eosin. Tumours were examined histologically and classified according to the World Health Organization (WHO) classification for tumours of domestic animals [26]. Carcinomas were classified by differentiation status [27] and surgical margins were evaluated to confirm that all masses were completely surgically ablated.

### Immunohistochemistry

IHC analysis was performed on 4 micron paraffin sections from all mammary tissue samples collected. Endogenous peroxidase activity was blocked with 3% hydrogen peroxide in methanol for 20 min at room temperature. The sections were subjected to high-temperature antigen unmasking by incubation with 98°C citric acid buffer (pH 6.0). Primary anti-human antibodies were used against mTOR (rabbit polyclonal antibody; catalogue number 2983, Cell Signaling Technology; diluted 1:50, overnight incubation), phospho-mTORSer2448-49F9 (rabbit polyclonal antibody; catalogue number 2976, Cell Signaling Technology; diluted 1:50, overnight incubation), c-erbB-2 oncoprotein (rabbit polyclonal antibody, Dako; 1:200 dilution; 1 h of incubation), PR (mouse monoclonal antibody, clone PR10A9 IgG2A, Immunotech Laboratories; 1:750 dilution, overnight incubation) and ERα (rabbit polyclonal, Zymed Invitrogen; 1:80 dilution; 1 h of incubation). Antibodies were detected with avidin-biotin peroxidase complex techniques using the Vectastain Elite ABC kit (Vector Laboratories). External positive controls were paraffin-embedded sections of MCF7 cells for mTOR and p-mTOR [28], a human breast carcinoma positive for HER2 and feline normal uterus for ERα and PR. Specificity of primary antibody was carried out incubating primary antibody with excess of specific peptide used to immunize rabbits (available from Cell Signaling Technology). After incubation the protein labeling was abrogated by the previous incubation of antibodies with the peptide.

The immunolabelled slides were randomised and masked for blinded examination, which was performed independently by three observers (LM, SI and YM); when there was disagreement (<5% of the slides), a consensus was obtained using a multi-head microscope. Slides with >20% tumour immunolabelled cells were considered to be positive for mTOR and phospho-mTOR [3]. HER2 immunoreactivity was scored according to the HerceptTest method (Dako), which is commonly used in human pathology and also adopted for FMCs [29]. ERα and PR positivity was evaluated as described previously [22].

### Cell lines and Western blot analysis

Six feline mammary tumour cell lines were evaluated: FYCp, P248m, FMCp, FMCm, FKNp and FNNm; the last four cell lines were kindly provided by Professor Sasaki, Japan [30]. P248m cell lines derived from a pulmonary metastasis of primary tubulopapillary mammary carcinomas [31] while FYC cell lines derived from tubulopapillary FMC [19,32,33]. Regarding cell lines kindly provided by Dr. Sasaki please see the reference [34]. The letters ‘p’ and ‘m’ indicate cell lines derived from primary and metastatic lesions, respectively. FYCp, FMCp, FMCm, FKNp and FNNm cells were grown in Roswell Park Memorial Institute (RPMI) medium, whereas P248m was grown in Dulbecco’s modified Eagle’s medium (DMEM). In all cases, the medium was supplemented with 10% fetal calf serum, 100 μg/ml penicillin, 100 μg/ml streptomycin and 1.5 mg/ml amphotericin B. For P248 cells, 10 μg/ml insulin was added to the DMEM.

Total protein were extracted at 80% of cellular confluence in boiling lysis buffer containing 1% sodium dodecyl sulphate (SDS) and 0.1 M Tris HCl (pH 6.8). Total proteins from each sample (40 μg) were separated on an 8% SDS-polyacrylamide (PAGE) gel and transferred onto a Hybond-C Extra membrane (Amersham Biosciences). Membranes were blocked at room temperature for 2 h with Tris-buffered saline (TBS, 10 mM Tris, 150 mM NaCl, pH 7.4) containing 10% bovine serum albumin, then incubated overnight at 4°C with the appropriate primary antibodies, including anti-ER, PR, HER2, mTOR (see antibodies used in IHC), phospho-mTORSer2448 (rabbit polyclonal antibody; catalogue number 2971; Cell Signaling Technology) or α-tubulin (mouse monoclonal antibody; catalogue number 3873; Cell Signaling Technology). After incubating with a horseradish peroxidase-linked secondary antibody that was diluted 1:2000 in TBS-Tween, the membranes were washed in TBS-Tween for 30 min and incubated with an enhanced chemiluminescence reagent (Super Signal West Pico Mouse IgG Detection Kit, Thermo Scientific). The proteins were visualised by exposing the membrane to a Hyperfilm ECL autoradiography film. Every experiment was carried out in triplicate and bands intensity was analyzed by using Biorad ChemiDoc XRS and ChemiDoc MP imaging system software.

### Statistical analysis

The IHC results were grouped into contingency tables and analysed using Fisher’s exact or *χ*2 tests. The DFI and OS analyses were performed using the Kaplan Meier method with a log rank test. P ≤0.05 was considered to be statistically significant. The data were analysed using GraphPad Prism Software v. 4.0.

## Results

### Clinicopathologic characteristics

The mean age of cats at the time of diagnosis was 11.32 ± 2.69 years (range 6–18 years). Of the 58 animals, 11/58 (19.0%) were spayed and 4/58 (6.9%) were cyclically treated with progesterone. At the time of diagnosis, histologically confirmed lymph node metastases were present in 5/58 (8.6%) FMC cases and no clinically and radiographically observable distant organ metastases were present. Of the 58 animals diagnosed with FMC, 13 (22.4%) did not present tumour recurrence and 45 (77.6%) relapsed 2–23 months after surgery. The mean DFI was 196.4 ± 153.1 days. Information regarding the OS was available for 25 cases within a 24 months follow-up period. Eighteen patients presented distant organ metastases during the follow up period and died of cancer-related causes confirmed at post-mortem examination.

The mean gross size of the tumours was 2.3 ± 1.8 cm and 15/58 (25.9%) tumours were ≤1.0 cm in diameter. All tumours were surgically completely ablated, which was confirmed histopathologically. A total of 39/58 (67.2%) tumours were tubulopapillary carcinoma, 11/58 (19.0%) were solid carcinoma, 7/58 (12.1%) were cribriform carcinomas and 1/58 (1.7%) was mucinous carcinoma. According to histological classification, 9/58 (15.5%) tumours were grade I, 24/58 (41.4%) were grade II and 25/58 (43.1%) were grade III. Out of the 18 benign mammary lesions used as controls, eight were lobular hyperplasia, five were fibroadenomas and five were simple adenomas.

### Immunohistochemistry

Immunoreactive products to ER monoclonal antibody in feline uterus used as positive control were observed in the nuclei of epithelial and stromal cells of the endometrium and the nuclei of smooth muscle myometrial cells. Immunoreactive products to PR monoclonal antibody in feline uterus used as control were observed in the nuclei of stromal cells and in the surface and glandular epithelial cells of the endometrium and of smooth muscle cells of the myometrium. In the normal mammary glands and in the mammary tumors object of this study immunoreactive products to ER and PR were observed in the nuclei of acinar and ductal glandular epithelial cells.

The human mammary carcinoma used as control for HER2 demonstrated a strong complete membrane staining in all the tumoral cells (HerceptTest 3+) while in feline mammary carcinomas, immunoreactive products were seen in the membrane and the cytoplasm of neoplastic cells and, according to HerceptTest method, only complete membrane staining was considered positive.

**Figure 1:**
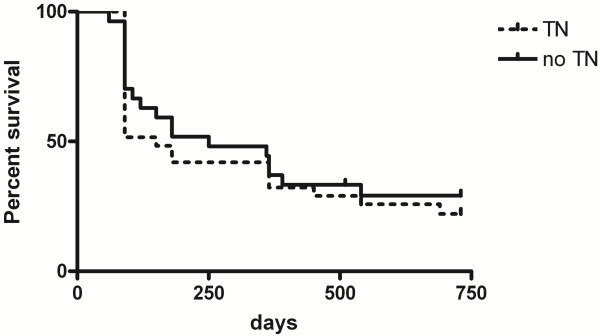
**DFI in patients with TN and no-TN feline mammary carcinomas.** Kaplan–Meier estimates for disease free interval probability in TN-FMCs (TN) and no TN_FMCs (no-TN) (P >0.05).

Based on the lack of the IHC expression of ER and the PR, as well as the lack of HER2 overexpression, 31/58 (53.4%) cases were TN-FMC and 27/58 (46.6%) were non-TN-FMC. Considering the immunophenotype of the steroid receptors, we found that 46/58 (79.31%) were ER-/PR-, 3/58(5.17%) were ER+/PR-, 6/58 (10.34%) were ER-/PR + and 3 were ER+/PR+. None of the feline benign mammary lesions, including the normal mammary tissue analysed, were of triple negative subtype. TN-FMCs compared to not TN-FMCs did not presented statistically significative differences in terms of clinicopathological features. The mean DFI observed in patients with TN lesions was shorter than the DFI reported in patients with non-TN cancer (150 days vs. 250) but not statistically significant (P >0.05) such as OS (Figure 1).

**Figure 2:**
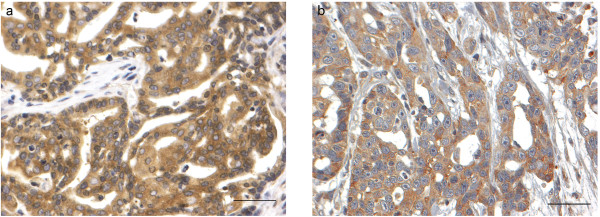
**mTOR expression in tubulopapillary carcinoma. a**. Mammary gland. I grade tubulopapillary carcinoma. Neoplastic cells are characterised by diffuse and moderate cytoplasmic immunopositivity for mTOR. Streptavidin-biotin-peroxidase method. Mayer’s haematoxylin counterstaining. Scale bar = 50 μM **b.** Mammary gland. III grade tubulopapillary carcinoma. Neoplastic cells are characterised by diffuse and moderate cytoplasmic immunopositivity for mTOR. Streptavidin-biotin-peroxidase method. Mayer’s haematoxylin counterstaining. Scale bar = 50 μM.

**Figure 3:**
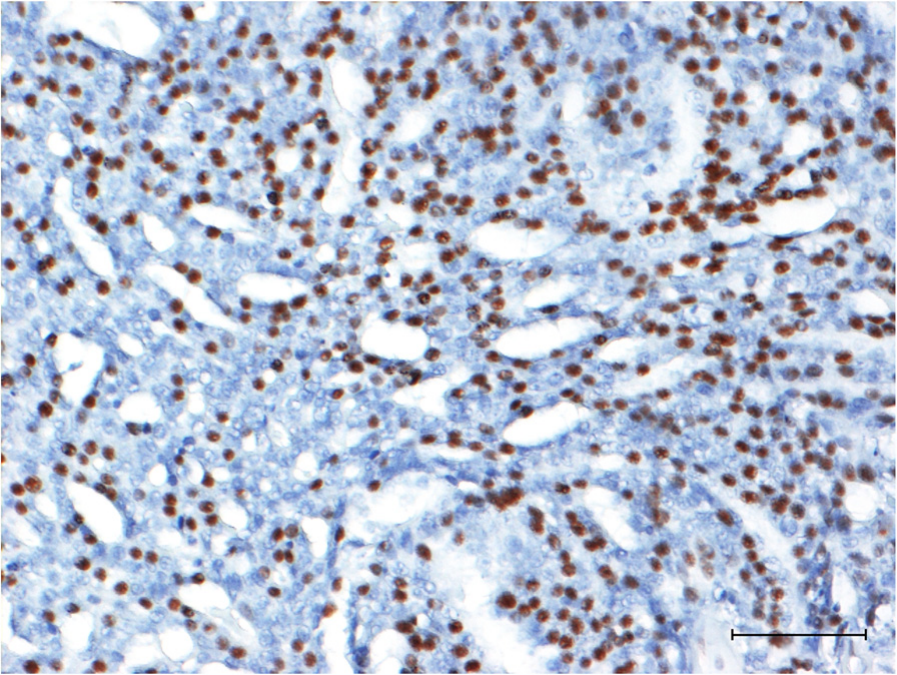
**- phospho-mTOR expression in tubulopapillary carcinoma.** Mammary gland. III grade tubulopapillary carcinoma. About 60% of neoplastic cells are characterised by strong nuclear immunopositivity for p-mTOR. Streptavidin-biotin-peroxidase method. Mayer’s haematoxylin counterstaining. Scale bar = 100 μM.

Immunoreactive products in paraffine-embedded sections of MCF7 cells, used as control for mTOR and p-mTOR, were seen in the 100% of the stained cells in the cytoplasm and in both the cytoplasm and nuclei respectively. Positive mTOR expression was detected in the cytoplasm of neoplastic cells by IHC analysis (Figures 2a and 2b) and was uniformly distributed within the tumour mass. Expression of p-mTOR was detected more frequently in the nucleus (Figure 3) and was also located in the cytoplasm. According to Walsh and colleagues [3], only the nuclear and peri-nuclear location was considered to be positive. When not diffusely located within tumour cells, p-mTOR was present in luminal epithelial cells. A statistically significant positive association was found between mTOR and p-mTOR expression (P <0.05). In benign lesions, three cases showed cytoplasmic positivity for mTOR (two fibroadenoma and one adenoma), while none of the benign lesions were p-mTOR positive. The normal feline mammary tissues were mTOR and p-mTOR negative.

Table 1 summarises the relationship between mTOR, p-mTOR, tumour characteristics and TN status. mTOR and p-mTOR were more frequently detected in TN compared with non-TN samples (P <0.05 for mTOR; P <0.001 for p-mTOR). There were no significant differences between the DFIs and OS analysis in relation to mTOR and p-mTOR expression. We also evaluated if the high expression of m-TOR and p-mTOR found in our samples was correlated to HER2, PR and ER expression expression. We found that samples HER2 negatives are statistically correlated to samples p-mTOR (P < 0.001) and m-TOR (P <0.05) positives (Table 1) while no correlations were found comparing mTOR and p-mTOR expression in relation to ER and PR phenotype.

**Table 1:** Relationships between tumour characteristics and expression of mTOR and p-mTOR

**Clinicopathologic characteristics**	**mTOR+**	**mTOR-**	***P***	**p-mTOR+**	**p-mTOR -**	***P***
	***n *(%)**	***n *(%)**		***n *(%)**	***n *(%)**	
Triple negative	22 (42.31%)	9 (17.31%)	0.03	24 (41.38%)	7 (12.07%)	0.0005
Non-triple negative	11 (21.15%)	16 (30.77%)		8 (13.79%)	19 (32.76%)	
Total	33	25		32	26	
			mTOR +	24 (41.38%)	8 (13.79%)	0.0032
			mTOR -	9 (17.31%)	17 (29.31%)	
				32	26	
			*			***
HER2 +	7 (12.07%)	12 (20.69%)	0.0479	4 (6.9%)	15 (25.86%)	0.0005
HER2 -	26 (44.83%)	13 (22.41%)		28 (48.28%)	11 (18.97%)	
Total	33	25		32	26	
>1 cm	27 (46.55%)	16 (27.59%)	>0.05	22 (42.31%)	20 (30.48%)	0.05
≤1 cm	8 (13.79%)	7 (12.07%)		10 (17.24%)	6 (10.34%)	
	35	23		32	26	
Grade I	5 (8.62%)	4 (6.90%)	>0.05	4 (6.90%)	5 (8.62%)	>0.05
Grade II	12 (20.69%)	12 (20.69%)		14 (24.14%)	10 (17.24%)	
Grade III	16 (27.59%)	9 (15.52%)		14 (24.14%)	11 (21.15%)	
Total	33	25		32	26	
Vascular invasion	7 (12.07%)	7 (12.07%)	>0.05	6 (10.34%)	8 (13.79%)	>0.05
No vascular invasion	26 (44.83%)	18 (31.03%)		26 (44.83%)	18 (31.03)	
Total	33	25		32	26	
Relapsed	25 (43.10%)	20 (30.48%)	>0.05	17 (29.31%)	28 (48.28%)	>0.05
Not relapsed	8 (13.79%)	5 (8.62%)		7 (12.07%)	6 (10.34%)	
Total	33	25		24	34	
Relapsed 0–6 months	17 (37.78%)	14 (31.11%)	>0.05	9 (20.00%)	22 (48.89%)	>0.05
Relapsed 6–12 months	4 (8.89%)	3 (31.03%)		4 (8.89%)	3 (31.03%)	
Relapsed >12 months	4 (8.89%)	3 (31.03%)		4 (8.89%)	4 (31.03%)	
Total	25	20		17	28	
Mitotic index^a^ 1	4 (6.90%)	2 (3.45%)	>0.05	3 (5.17%)	3 (5.17%)	>0.05
Mitotic index 2	7 (12.07%)	8 (13.79%)		8 (13.79%)	7 (12.07%)	
Mitotic index 3	22 (42.31%)	15 (25.86%)		21 (36.21%)	16 (27.59%)	
Total	33	25		32	26	

^a^Mitotic index of the histological grading system [27].

### Western blot analysis

**Figure 4:**
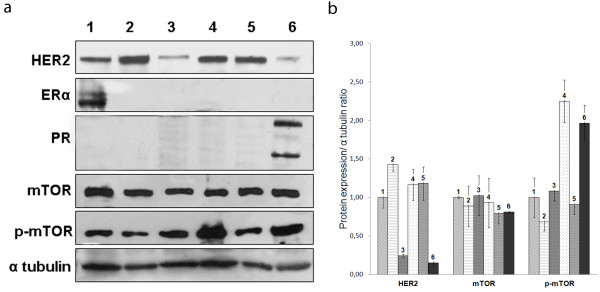
**Western blot. a**. Western blot analysis of HER2, oestrogen receptor α (ERα), progesterone receptor (PR), mTOR and p-mTOR expression in FYCp (lane 1), P248m (lane 2), FMCp (lane 3), FMCm (lane 4), FKNp (lane 5) and FNNm (lane 6) cell lines. α Tubulin expression was used as the loading control. **b**. Measure of band intensity normalized to α tubulin. Error bars demonstrate standard deviation.

To validate the antibodies used in immunohistochemistry and to further investigate mTOR expression in TN-FMC, Western blot analysis of mTOR, p-mTOR, ER, PR and HER2 was performed on FMC cell lines (Figure 4). A specific 289 kDa band corresponding to human mTOR was found in all cell lines analysed. A specific 286 kDa band corresponding to phospho-mTORSer2448-49F9 was visible in all cell lines, with the strongest expression in FMCm and FNNm. A band corresponding to ERα (67 kDa) was also present in the FYC cell line, while specific 116 kDa (B form) and 81 kDa (A form) double bands corresponding to PR were present in the FNNm cell line. HER2 band (185 kDa) was detected in all of the FMC cell lines. The FKNp, FMCm, P248m and FYCp cell lines demonstrated higher expression of HER2 than the FYCp, FMCp and FNNm cell lines; cell line derived from the metastatic tumor (FMCm) shows a higher expression of HER2 compared to the cell line derived from the primary tumor (FMCp).

## Discussion

This study provides the first evidence that felines also have a subset of mammary carcinoma that is defined by the lack of immunohistochemical ER and PR expression and a lack of HER2 overexpression, which we identify as TN FMC. Similar to human breast cancer, these tumours demonstrate high expression of mammalian target of rapamycin (mTOR). Human TNBCs have a poor prognosis, because such cancers have no effective therapeutic targets, e.g. ER for endocrine therapy or human epidermal growth factor receptor 2 receptors for anti-HER2 therapy [35-37]. For these reasons, several efforts are underway to better characterize this type of tumour to develop new targeted therapies and to identify a new animal model for comparative oncology.

Among spontaneous tumours occurring in domestic animals, feline mammary tumour is widely considered an excellent model for human breast cancer. In particular, the high percentage of FMCs negative for ER and PR, ranging from 37% [20] to 54.2% [21], makes this tumour a suitable model of breast cancer hormone independent subtype [24]. Moreover we and others [19,22,23] showed that HER2 is expressed from 39% to 56.3% and is a negative prognostic factor [23] similar to human breast cancer (20-30%) [24] while Rasotto and colleagues [25] demonstrated that only the 5.5% of FMCs was HER2 positive and it is not a prognostic factor in FMCs. This discrepancy might be due to the use of different antibodies, criteria of evaluation and other issues related to discordant study protocols [38] and, considering the potential importance of FMCs as model for human breast cancer, a standardised method for the detection of HER2 expression and cellular localisation in feline mammary tumours is urgently needed. In addition, our group has demonstrated that p-AKT is expressed in a high percentage of FMCs and correlated to poor prognosis [22], suggesting that the PI3K/AKT/mTOR pathway is activated and associated with oncogenesis in the feline species, which is similar to that in humans [14] and recently also demonstrated in canine tumour cells [39] These assumptions prompted us to better understand the involvement of mTOR in FMC.

The IHC results obtained in this study demonstrated that there is a FMC tumour subtype that we can characterise as TN FMC since 53.4% of feline FMCs do not express ER and PR and also lack HER2 overexpression. These data are consistent with the description of human TNBCs [1] even if more expressed and confirm the presence of a new entity in feline mammary oncology.

As previously described, one of the promising human TNBC therapies is the inhibition of the PI3K/AKT/mTOR pathways, particularly mTOR, which is more frequently expressed in TN cancer compared with its non-TN counterparts [3] and is considered to be a potential anticancer therapy molecular target [9,13-15]. We have previously shown that the PI3K/AKT/PTEN pathway was activated in feline mammary carcinoma and correlated with poor prognosis [22], while there are no scientific data demonstrating mTOR expression in FMC tissues and cell lines with respect to the TN phenotype and AKT activation as demonstrated in humans. To elucidate whether feline mTOR was also phosphorylated by AKT, we evaluated both mTOR and p-mTOR at serine 2448, which represents a specific p-AKT phosphorylation site.

In this study, we observed that the 56.9% and 55.2% of FMC samples expressed mTOR and p-mTOR respectively and we found a statistical association between mTOR and p-mTOR expression in the same samples (P <0.005) analysed. These data suggest that the mTOR protein is widely present in feline mammary carcinomas in its Ser2448 phosphorylated form, which is regulated by AKT [6]. These results are similar to human breast cancer, in which mTOR is expressed in 47% of cases [3] while p-mTOR expression has been reported in 24% to 69.7% of cases [14,40].

The correlation between mTOR and p-mTOR expression and prognosis, tumour histological grading, vascular invasion, tumour size and mitotic index did not demonstrated any statistically significant correlations, suggesting that neither mTOR nor its putative active form (p-mTOR) have a prognostic role in feline mammary carcinoma. In human breast cancer, the prognostic significance of p-mTOR expression is still controversial [3,14,41]. With regard to expression in TN-FMC, this study observed that mTOR and p-mTOR expression are statistically associated with the TN phenotype. This evidence is consistent with the results described in humans by Walsh and colleagues [3] that observed a higher expression of p-mTOR in TNBCs. As shown in Table 1 we investigated if the high expression of m-TOR and p-mTOR founded in our samples was correlated to HER2 expression and we found that samples HER2 negatives are highly correlated to samples p-mTOR (P = 0.0005) and m-TOR (P = 0.0479) positives. These data confirm as in human [3] that p-mTOR is statistically correlated to triple negative phenotype. Interestingly, we found an opposite correlation between HER2 expression and mTOR phosphorylation. As during progression of cancers selection operates favouring cells with activated oncogenes, it is not surprising that cells showing the activation of a HER2 downstream signal, such as mTOR, do not require the simultaneous activation of the upstream signal, i.e. HER2. This for example also occurs in cells showing either RAS or RAF mutations, which are mutually exclusive in colorectal cancer [42] and melanoma [43].

As described in results, western blot was performed mainly to identify the specify of antibodies and the corresponding proteins expression in feline mammary cells lines. The expression of PR, ER, HER2, mTOR, p-mTOR was confirmed by immunocytochemistry on each cellular monolayer (data not shown). On the basis of western blot analysis we can assess that 4/6 cell lines are negative for PR and ER, suggesting the non hormonal-dependent behavior of feline mammary carcinomas. Only FMCp cell line resulted ER and PR negative and with a low level of HER2 showing a triple negative behavior. All cell lines demonstrated high mTOR and p-mTOR expression with a higher expression of p-mTOR in the FMCp, FMCm and FNNm cell lines. The higher mTOR phosphorylation in the metastasis-derived cell line (FMCm) compared with its primary counterpart (FMCp) suggests a significant involvement of p-mTOR in metastasis, invasion and FMC tumor progression, such as in human breast cancer [14,44].

mTOR is considered a potential target for anti-tumor therapies [14,44] due to its role in breast cancer progression. The finding shown here, demonstrating the strong activation of mTOR in triple negative FMC, suggests that this tumor may be a suitable model to test innovative therapies against the human TNBCs.

## Conclusions

Spontaneous tumours occurring in companion animals represent a significant resource for models to study innovative therapies for human cancer and feline mammary carcinoma is considered a suitable model for human hormone independent breast cancer. In this study, we demonstrate that there is also a FMC subset defined as TN FMC, which is characterised by a statistically significant association with m-TOR and p-mTOR expression as demonstrated in human breast cancer.

## Competing interests

The authors declare that they have no competing interests.

## Authors’ contributions

LM: participated in the design of the study, performed histological diagnosis, immunohistochemical interpretation, western blot analysis and drafted the manuscript. YM, SI performed: histological diagnosis and immunohistochemical interpretation, MD carried out the immunohistochemistry. RSC: provided technical support FG: provided follow-up and clinical data of the patients. BB: gave scientific support. NS, TN: provided feline mammary cell lines. MFDR: gave scientific support. JMM participated in the design of the study. RDM: conceived the study, participated in its design and coordination and helped to draft the manuscript. All authors read and approved the final manuscript.
